# Efficacy of radiographic density values of the first and second cervical vertebrae recorded by CBCT technique to identify patients with osteoporosis and osteopenia

**DOI:** 10.15171/joddd.2017.034

**Published:** 2017-09-20

**Authors:** Farzad Esmaeli, Salar Payahoo, Majid Mobasseri, Masoome Johari, Javad Yazdani

**Affiliations:** ^1^Department of Oral and Maxillofacial Radiology, Faculty of Dentistry, Tabriz University of Medical Sciences, Tabriz, Iran; ^2^Endocrinology and Metabolism Section, Department of Medicine, Imam Reza Hospital, Tabriz, Iran; ^3^Department of Oral and Maxillofacial Surgery, Faculty of Dentistry, Tabriz University of Medical Sciences, Tabriz, Iran

**Keywords:** CBCT, osteoporosis, cervical vertebrae, radiography

## Abstract

***Background.*** Osteoporosis is a systemic skeletal disease characterized by a decrease in bone strength with an increase in the risk of fractures. This study aimed at evaluating the ability to predict osteoporosis and osteopenia based on radiographic density values obtained from CBCT imaging technique.

***
Methods.*** CBCT images of 108 patients were prepared by using NewTom VGI (QR, Verona, Italy). Then the patients were assigned to osteoporosis, osteopenia and healthy group, using the T-score derived from the DEXA technique. Finally, RD of the lateral mass of C1 on the left and right sides and body and dens of the C2 were measured. RD values were compared between the three groups by one-way ANOVA, followed by an appropriate post hoc test.

***
Results.*** The results of the comparisons of RD values at the first and second cervical vertebrae in the three groups showed that all the values had statistically significant differences (P<0.05). The most precise diagnosis of osteoporosis was related to the RD values of the body of C2 and left lateral mass of C1 that was equal to 99% and their cut-off points were 375 and 386, respectively.

***
Conclusion.*** Based on the findings of this study, it is possible to predict the osteoporosis status of the patient through the RD related to the body of C2 and the left lateral mass of C1 more accurately than the other areas.,

## Introduction

Osteoporosis is a systemic skeletal disease that leads to loss of bone density and an increase in fracture risk.^1^ It causes high mortality and economic costs (over 13 billion dollars in the USA); for this reason, it has attracted great attention.^2,3^ Based on epidemiological studies, more than 100 million people over 50 years of age in America and about 28% of women over 50 years of age in Iran have osteoporosis and 53% have osteopenia.^4-6^

This disease leads to a reduction in bone density and quality of microstructures, leading to an increase in bone fragility.^7^ For this reason, determining bone mineral density (BMD) values does not consider all the factors affecting bone strength.^8^ According to WHO criteria, T-score is used for expressing the status of individuals in terms of developing this disease, so that people with T-scores ≥-1 are considered healthy, those with -1> T-score> - 2.5 are considered osteopenic and those with 2.5≥ T-score are considered osteoporotic.^9^

Fractures that occur in the lumbar spine due to this disease often lead to chronic pain (especially in standing position) in the back area and affect the patient's quality of life (6). Conversely, in patients with painful vertebral fractures, the mortality rate increases by 15%; therefore, early detection of the disease enhances its prognosis.^10,11^ Dual-energy x-ray absorptiometry (DEXA) is the most common technique for obtaining bone mineral content (BMC) and BMD which is often used in the assessment of central bone such as the lumbar spine and hip.^12-14^ Recently, cone-beam computed tomography (CBCT) technique has been introduced to dentistry, which is able to provide 2D and 3D images and has tools for measuring bone density. The advantages of this technique when compared to multi-detector computed tomography (MDCT) are reduced cost and low patient exposure.^15,16^

However, despite the ability to detect bone density and the quality of structures in this technique, few studies have employed this technique to assess osteoporosis.^17^ In previous studies, a strong relationship has been found between the BMD of the mandibular cortical bone and cervical vertebrae in DEXA technique.^18^ Also, a positive relationship has been found between the BMD of mandibular bone and the lumbar spine in patients with osteoporosis.^19^ Therefore, we can probably argue that there is a relation between the BMD of lumbar spine and cervical vertebrae. Furthermore, in another study, Brangkegi et al determined RD values of dens (the odontoid process of the second cervical vertebra) of C2 and lateral mass of the left side of the C1 with the use the CBCT technique and showed high sensitivity and accuracy in predicting the risk for individuals with osteoporosis.^20^ Given that a large number of people are affected by osteoporosis, dental radiographs of patients can be used as a tool for screening it.^21,22^ Several studies have used the radio morphometric index of the mandible and fractal dimensions in CBCT for evaluating the bone status of patients but only one study (Brangkegi^20^) has used the BMD derived from CBCT to assess bone status. The aim of this study was to assess the ability of CBCT technique to predict the risk of developing osteoporosis and help in the early detection with this device and software and to compare it previous studies with larger statistical populations.

## Methods

This cross-sectional study was conducted in 2014‒2015 on 58 patients with osteoporosis and osteopenia in the lumbar spine, with age range of 42‒72 years. Furthermore, 50 individuals without any systemic disease, who were treated by densitometry with T-cores in the lumbar spine in the normal range, were considered as the control group. All these steps were conducted in collaboration with and under the direct supervision of an endocrinologist. Individuals with diabetes, thyroid disorders, bone diseases except for osteoporosis, alcohol users, cigarette smokers and those taking drugs that affect BMD and individuals with a history of fracture of the femoral neck and lumbar spine were excluded from the study. In this study, in order to avoid additional costs and the damaging effects of x-rays, CBCT images of patients requiring preparation of stereotypes for dental treatment and those who had densitometry history in the past year by the DEXA scanner (Hologic QDR 4500/Acclaim, USA) were used.

Then the subjects were divided into three groups of osteoporosis, osteopenia and healthy according to WHO criteria and based on T-score of the lumbar region. Finally, the relationship between the RD values of C_1_and C_2_ was evaluated with T-score in the lumbar spine.

CBCT images were provided by using NewTom VGI (QR, Verona, Italy), in FOV (15*12 cm^2^) at amorphous silicon flat panel detector with effective dose (86 μSv), voxel size of 0.2 mm, and focal spot size of 0.3 mm. This system uses rotating anode: 110 kVp and 1‒20 mA, and takes images at 360 rotations and the scan time is 18 s. All the exposure parameters were set automatically. Then, these images were observed by NNT Viewer software on a 19-inch Philips 190B LCD (Philips, Eindhoven, Netherland) with a resolution of 1024×1024 and 16 bit in a room with a dim light and without any windows. Furthermore, all the principles of radiation protection such as the use of a lead apron were observed for all the subjects. In order to show that there was no difference in the RD measurement between different scans, the homogeneity of RD was tested between scans by using distilled water and this procedure was repeated during the scanning of all the patients. According to the device’s bit depth, differences in RD rate of water, which was achieved by using a measurement method of Spin-Neto et al,^23^ indicated high homogeneity in scan densities.

**Figure 1:**
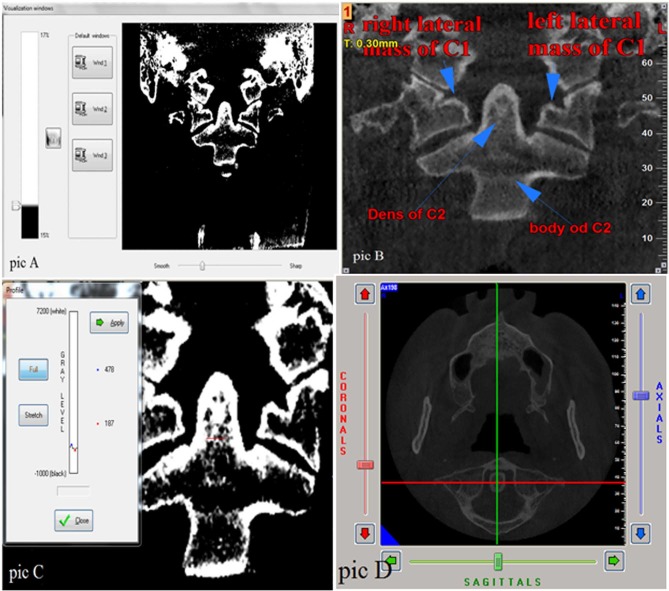
A, Adjusting the window width, window level and sharpness of the images. B, Areas under review. C, Measuring RD values in dens. D, The coronal section passes through the middle of the dens.

These steps lead to the creation of a homogenous density between different scans and enhancement of credibility of the present study. RD values were recorded in window width of 17% and window level of 15%, which make the images white and black. Also the sharpness was adjusted to provide an approximately smooth image (Figure 1-A). Then the RD values were recorded in four areas, including lateral mass of C_1_ on the both the right and left sides and the dens and body of C_2_ by using NNT Viewer software (Figure 1-B, C). The coronal section which passes through the middle of the dens (Figure 1-D) was selected and evaluated under a magnification of %175. RD values were recorded in five sites of these areas; four of the sites were in the margins and one of them was in the center of the areas. Then, the average of RD values was calculated and this number was considered as the area’s main RD value.

### Statistical analysis

The data obtained from this study were reported by using descriptive statistical methods (mean ± SD). RD values were compared between the three groups by one-way ANOVA and appropriate post hoc tests were used. In cases in which the result of Levine’s test was significant, post hoc Tukey test was used and in case of being non-significant for this test, post hoc Games-Howell test was used. Kolmogorov-Smirnov test was used to evaluate the normality of data. Also in order to evaluate the prediction of the risk of osteoporosis and osteopenia accurately by these values, NPV and PPV indexes and the sensitivity and specificity criteria and cut-off points were reported for each area.

## Results

Age-related features in different groups, including the healthy group, osteopenia and osteoporosis, are presented in Table 1. Also, the descriptive data regarding the RD values are classified for healthy individuals, osteopenia and osteoporosis in Table 2. The results presented in Table 1 show that the average age in the osteoporotic group was the highest (63.2 years) and the group of healthy individuals exhibited the lowest (48.5) (Table 1).

**Table 1:** Mean ages of participants in three different groups (n=108)

**Group**	**age**
**Healthy (n=50)**	**48.5 (5.6)**
**Osteopenia (n=36)**	**56.3 (6.8)**
**Osteoporosis (n=22)**	**63.2 (5.4)**

Data presented as Mean (SD)

**Table 2:** RD values for healthy individuals, osteopenia and osteoporosis [Mean (SD)]

**Variables**	**Osteoporosis (22 persons)**	**Osteopenia(36 persons)**	**Healthy (50 person)**
**Left lateral mass of C**_1_	222 (56)	354 (106)	424 (71)
**Right lateral mass of C**_1_	245 (26)	340 (96)	424 (71)
**Body of C2**	224 (19)	300 (60)	416 (55)

All numbers are rounded and written without decimals.

RD values of the first and second cervical vertebrae are presented in Table 2 separately for the group of healthy individuals and those with osteopenia and osteoporosis. As expected, these values were the highest in healthy individuals and the lowest in patients with osteoporosis, respectively. In addition, the results of the comparisons of RD values showed that all the values in the three groups were significantly different from each other (P<0.05).

Finally, in evaluating the mean difference between the two groups, it was established that there was no significant difference except for that between dens values in the group of patients with osteopenia and osteoporosis (P=0.379) and this difference was significant between other variables in the three groups (P<0.05).

Table 3 presents the reliability of RD values to predict to what extent individuals are affected by osteoporosis. It was established that the highest accuracy in predicting osteoporosis was obtained by RD values of the body of C_2_ and the left lateral mass of C_1_ and this accuracy was estimated at 99% and their cut-off points were 375 and 386, respectively‏. The lowest accuracy in predicting osteoporosis was related to RD values of dens with accuracy of 72% and cut-point of 544 (Table 3).

**Table 3:** Credit of RD values as a tool to predict affection by osteoporosis

**Area**	**Variable**	**AUC (95% CI)**	**Cut-off value**	**Sen**	**Spe**	**PPV**	**NPV**
**Lumbar spine**	Left lateral mass of C_1_	0.99 (0.98-1)	386	92%	100%	100%	64%
	Right lateral mass of C_1_	0.98 (0.95-1)	350	98%	100%	100%	73%
	Body of C2	0.99 (0.99-1)	375	100%	100%	100%	70%
	Dens of C2	0.72 (0.59-0.84)	544	68%	69%	79%	46%

Positive and negative predictive values showed the possibility of being ill if the test answer is positive and possibility of being healthy if the test answer is negative. The results indicated that the highest positive predictive value relating to the right and left lateral masses of C_1_ and body of C_2_ was 100% in the lumbar spine; this implies that 100% of individuals whose test answer was positive through these RD values were ill‏. Also, the highest negative predictive value relating to the right lateral mass of C_1_ RD values according to the T-score of lumbar spine had a probability value of 73%, indicating that the probability of an individual being healthy would be 73% if the test answer is negative.

Table 4 provides the reliability of RD values to predict to what extent individuals are affected by osteopenia. As can be observed, the highest accuracy for predicting osteopenia in the lumbar spine was obtained through RD values of the body and this accuracy was 91% and its cut-off point was 375. The lowest accuracy in this prediction was related to dens RD values with accuracy of 66% and its cut-off point was 533. In this table, the results show that the highest positive predictive value relating to body values in lumbar spine was 91% and this implies that 91% of people whose test answers were positive through these RD values were ill. Moreover, the highest negative predictive value relating to the right C_1_ RD values according to the T-score of lumbar spine with a probability of 78% showed that the probability of an individual being healthy would be 78% if the test answer is negative.

**Table 4:** Credit of RD values as a tool to predict affliction with osteopenia

**area**	**Variable**	**AUC (95% CI)**	**Cut-off value**	**Sen**	**Spe**	**PPV**	**NPV**
**Lumbar spine**	Left lateral mass of C_1_	0.76 (0.65-0.87)	386	76%	78%	82%	70%
	Right lateral mass of C_1_	0.78 (0.67-0.9)	350	84%	78%	83%	77%
	Body of C2	0.91 (0.85-0.98)	375	84%	89%	91%	78%
	Dens of C2	0.66 (0.54-0.78)	533	76%	64%	72%	60%

## Discussion

Currently, osteoporosis is one of the main challenges of communities and organizations associated with health in most countries. Since life expectancy is increasing in most countries, it is estimated that people over 65 years of age will increase from 323 million to over 1.5 billion by 2050, indicating that fractures caused by osteoporosis will increase from 1.6 million in 1990 to 6.26 million by 2050.^24,25^

Of the debilitating and life-threatening complications of this disease, hip fracture can be mentioned in a way that a mortality rate of 10‒20% has been reported in the first year after its occurrence.^26^

The current study suggests that by using the RD values relating to body of C_2_ and the left lateral mass of C_1_, the status of osteoporosis in the lumbar spine can be predicted in individuals undergoing CBCT imaging.

Such studies can play an important role in the early diagnosis of osteoporosis before imposing heavy financial costs. Therefore, this study increases the possibility of diagnosis of patients with osteopenia as individuals who are in the middle limit of health and osteoporosis. However, few studies have been conducted on the use of RD values in predicting osteoporosis, so it cannot be argued with certainty about the predictive values of RD.^27,29^ Software used in this technique has the tools needed for basic analysis such as multi-planar reconstruction, measurement of dimensions, calculation of RD and calculation of mean values of voxel. Despite the numerous advantages of CBCT scanner, this technique has defects in calculating the RD.^27^ Increases in noise and value of beam scattering, especially when the size of the voxels is small, the divergence phenomenon of cone beam, low efficiency of detector and artifacts associated with scanner result in a decrease in the technique accuracy of CBCT in the calculation of RD.^28,30,31^

Barngkgei et al^20^ evaluated the ability of CBCT in detecting osteoporotic patients. It was established that RD values relating to the dens of C_2_ and left lateral mass of C1 were highly accurate in the prediction of osteoporosis.

Based on the results of the present study, RD values relating to the Body area of C2, with prediction accuracy of over 99% and cut-off point of approximately 375, had a great predictive value for osteoporosis. The discrepancies between our study and the above-mentioned study^20^ might be related to the use of different devices, software programs and different exposure parameters that affect RD value assessments. In addition, the number of people surveyed in our study was (108) more than that in Barangkegi’s^20^ study (38).

Generally, the signal-to-noise ratio in CBCT scanner is less than that in MDCT that is the gold standard and varies between devices.^32^ Furthermore, there are many differences between the RD values calculated with various devices and also different exposure conditions in a device.^33,34^ Thus, RD values derived from our device might not match those in other CBCT devices. In order to confirm the results of this study, it is better that this study be conducted with larger sample sizes, regarding the credibility of RD values derived from the CBCT of cervical vertebrae to predict osteoporosis at different ages. Finally, it is advisable to use this technique to screen osteoporosis alongside the main applications.

## Conclusion

Based on the results of this study, it is possible to predict the osteoporosis status of the patient through the RD values related to the body of the C2 and left lateral mass of the C1 more accurately than the other areas.

## Acknowledgements

The authors thank all our friends and members of staff of Oral and Maxillofacial Department and Sina Hospital, for their cooperation in conducting the study.

## Authors’ contributions

The concept and the design of the study were developed by FE, MJ and SP. The acquisition, analysis and interpretation of data were accomplished by FE, MM, JY and SP. All the authors participated in the literature review. FE, MM, MJ and SP drafted the manuscript and all authors revised it critically for intellectual content. All authors have read and approved the final manuscript.

## Funding

This study was financially supported by Dental and Periodontal Research Center in Tabriz University of Medical Science.

## Competing interests

The authors declare no competing interests with regards to the authorship and/or publication of this article.

## Ethics approval

The study protocol was approved by the Research Ethics Committee of Tabriz University of Medical Sciences.
