# Correction to: Efficacy of radiographic density values of the first and second cervical vertebrae recorded by CBCT technique to identify patients with osteoporosis and osteopenia

**DOI:** 10.15171/joddd.2018.048

**Published:** 2018-12-19

**Authors:** Farzad Esmaeili, Salar Payahoo, Majid Mobasser, Masoome Johari, Javad Yazdani

**Affiliations:** ^1^Dental and Periodontal Research Center, Tabriz University of Medical Sciences, Tabriz, Iran; ^2^Department of Oral and Maxillofacial Radiology, Faculty of Dentistry, Tabriz University of Medical Sciences, Tabriz, Iran; ^3^Endocrinology and Metabolism Section, Department of Medicine, Imam Reza Hospital, Tabriz University of Medical Sciences, Tabriz, Iran; ^4^Department of Oral and Maxillofacial Surgery, Faculty of Dentistry, Tabriz University of Medical Sciences, Tabriz, Iran

In the article entitled "Efficacy of radiographic density values of the first and second cervical vertebrae recorded by CBCT technique to identify patients with osteoporosis and osteopenia" which appeared in *J Dent Res Dent Clin Dent* Prospect 2017; 11(3):189-194, the name of the first author was misspelled. The correct name of the first author is Farzad Esmaeili. The authors’ affiliation with Dental & Periodontal Research Center of Tabriz University of Medical Sciences was also missing from the article. The original version of the article has been updated to reflect these corrections.

